# The effect of home-based cardiac rehabilitation program on self efficacy of patients referred to cardiac rehabilitation center

**DOI:** 10.1186/1756-0500-6-287

**Published:** 2013-07-23

**Authors:** Sarieh Poortaghi, Ali Baghernia, Samad EJ Golzari, Abdolrasoul Safayian, Shirin B Atri

**Affiliations:** 1 Faculty of Nursing & Midwifery, Tehran University of Medical Sciences, Tehran, Iran; 2 Tabriz University of Medical Sciences, Tabriz, Iran; 3 Cardiovascular Research Center, Tabriz University of Medical Sciences, Tabriz, Iran; 4 Students’ Research Committee, Tabriz University of Medical Sciences, Tabriz, Iran; 5 Health & Nutrition Faculty, Tabriz University of Medical Sciences, Tabriz, Iran; 6 Nursing & Midwifery Faculty, Tabriz University of Medical Sciences, Tabriz, Iran

**Keywords:** Rehabilitation, Heart disease, Self-efficacy

## Abstract

**Background:**

The prevalence of coronary heart disease is increasing worldwide contributing to mortality and morbidity of millions of people. Cardiac rehabilitation is an interdisciplinary activity with the aim of facilitating and improving the physical, psychological and emotional state of the patients with cardiac complications. This study aimed to evaluate the probable positive effects of continuing cardiac rehabilitation programs at home on self-efficacy of the patients with cardiac complications.

**Method:**

In a randomized controlled trial, 80 patients referred to rehabilitation center from Feb 2009 to Jan 2010 were randomly divided into case and control groups. Both groups received routine cardiac rehabilitation programs in the rehabilitation center. In addition, the case group received education and practical training in various rehabilitation measures along with home visits of a community health nurse throughout the follow-up period. General Self Efficacy Scale (GSES) was used to assess self-efficacy between two groups at baseline and follow-up periods. Collected data from GSES questionnaires were analyzed using Minitab software and repeated measurement analysis model.

**Results:**

No first time (before beginning of rehabilitation program) GESE differences were observed between case (26.36 ± 0.84) and control (28.53 ± 0.54) groups (P = 0.44). In the final measurement, GESE in case group was 36.59 ± 5.65 vs. 26.5 ± 0.91 in the control group. There was a statistically significant difference in self-efficacy between two groups and in different measurements (P = 0.000).

**Conclusion:**

Home-based cardiac rehabilitation has a positive effect on patients’ self-efficacy and therefore it is recommended for the patients suffering from heart diseases.

**Trial registration:**

RCT registration number: IRCT201106086747N1.

## Background

Coronary Heart Disease (CHD) is considered as an important source of disability and economic burden to health care systems globally. It is also known as one of the major causes of premature death. The high prevalence of CHD is believed to correlate strongly with the lifestyle and altered physiological factors. Modification of these risk factors has been shown to considerably reduce mortality and morbidity [1].

Cardiovascular disorders, mostly considered as an epidemic, will have contributed to immense morbidity, disability and economic burden by 2020 [2]. In recent decades, thanks to the improved diagnostic methods and successful treatments, the mortality rate from the disease has reduced in many developed countries; however, the morbidity rate has increased [2,3]. A comparison between the studies in the U.S and Europe with Iran has shown a similar pattern of prevalence and risk factors of the disease. Cardiac rehabilitation, as an evidence based intervention which aims to optimize patients functioning, enhances the quality of life and minimizes the risk of recurrent cardiac events [4]. Recent systematic reviews and meta-analyses [1], and [5] suggest that patients who participate in cardiac rehabilitation programs would benefit significant reductions in mortality and morbidity (i.e. improvement in exercise tolerance, symptoms, blood lipid profiles, blood pressure and psychosocial well-being).

Home-based programs have been developed to provide nurse-led, community-based, self-help programs for patients who may not be able to repeatedly attend hospital-based programs. With the increasing financial burden of coronary heart disease worldwide, the development of an affordable, acceptable and appropriate method of community-based cardiac rehabilitation is of significant importance [6].

Recently, home rehabilitation for patients with cardiac diseases following uncomplicated heart attack has been developed as a model of care focusing on the fact that responsibility of client is appointed to him/herself thus resulting in increased independence [7,8].

Numerous studies have been published comparing home-based cardiac rehabilitation with supervised centre-based cardiac rehabilitation programs which have been associated with variable results [2,9-12]. Some of these studies report similar improvements in exercise capacity, and systolic blood pressure and serum cholesterol levels in follow-up periods in both home and centre-based groups. In a study carried out by Dalal et al. in 2010, no significant difference was observed in short term (3–12 months) or long term (up to 24 months) outcomes between patients with stable coronary heart disease who received home-based vs. centre-based cardiac rehabilitation. However, significantly reduced hospital admissions were reported in the home-based group during the first 6 months of the follow-up period compared to the patients having received usual care.

In a cardiac rehabilitation program following CABG the patients in the home-based arm reported a significantly improved quality of life compared to patients attending a hospital program [10].

There are few drawbacks of centre-based rehabilitation. The main reasons people give for not accepting the invitation to attend centre-based cardiac rehabilitation classes—held for groups in hospitals, gyms, or community leisure centers—are problems with accessibility and parking at their local hospital, a dislike of groups and work or domestic commitments. These problems can be overcome by home-based programs which have been introduced in an attempt to widen access and participation of the patients [4].

Home-based rehabilitation of the patients is conducted by house visits of the community health nurses. Performing as coordinators and/or performers of the rehabilitation programs, community health nurses act in three levels of prevention. They also play an important role in assisting patients with restarting their functions or increasing their activities and exercise during a cardiac rehabilitation program in a hospital or institute [13].

The aim of this study was to evaluate the probable positive effects of continuing cardiac rehabilitation programs at home on participants’ self-efficacy.

## Methods

This randomized controlled trial was conducted in Tabriz Shahid Madani rehabilitation center, Tabriz, Iran from Feb 2009 to Jan 2010. The study was approved by the ethics commitee of Tabriz University of Medical sciences and all patients gave their oral and written informed consent to participate. Study population included all patients referred to the ambulatory rehabilitation center 45 days after being discharged from hospital. Patients were placed in the groups of post-CABG, MI and PTCA. Baseline assessments including echocardiography and exercise tolerance test were performed by a cardiologist who was blinded to the study. Inclusion criteria were age between 30–75 years, lack of mobility limiting diseases, mental disorders, uncontrolled heart failure or arrhythmias and stable angina. Patients not willing to participate in the study or having developed any cardiovascular or musculoskeletal complications during rehabilitation program were excluded from the study. Sample size based was estimated as a total of 80 patients based on previous studies [14,15] with confidence of 95%, power of 95%, standard deviation of 1.75, minimum Distance Separation of 1.2 and Cohen’s d effect size of 0.78.

All patients meeting the inclusion criteria and giving informed written consent were randomly allocated to the intervention or control groups.

GSES (General Self-Efficacy Scale) questionnaires, a valid and reliable scale been used in several studies in Iran [16,17], were given to both groups to be completed.

GSES, containing 17 items, is used to detect self-efficacy in the general population as well as general medical outpatients. All items have a spectrum scoring system from zero to 3. The score of general self-efficacy has a direct relation with the values in the table.

Both groups received routine cardiac rehabilitation program in the rehabilitation center and at the end of the 12th session, GSES questionnaires were completed by all of the patients. In the case group, education regarding risk factors, nutrition, taking medication and the necessity of continuing program at home was given by the nurses and a nutritionist. Practical training about measuring heart rate, detecting target heart rate, doing suitable exercises at home, setting the home exercise program, walking and jogging was performed by a team consisting of nurses and a physiotherapist. Furthermore, the structure and the contents of the training course were handed out to the patients in the intervention group.

At the end of the first and second months of the follow-up program, researcher, as a community health nurse, visited patients in the case group twice at home. At home visits the nurse controlled the continuity of rehabilitation program and discussed possible problems. In both home visits, GSES questionnaires were completed. Meanwhile, GSES questionnaires were completed in the control group and the required data were collected. As previously mentioned, this study was a repeated measurement and GSES questionnaires were completed for four times by each patient. At the end of the study, due to ethical considerations, written and practical education was provided for the control group.

After data collection, demographic characteristics were analyzed using descriptive statistics including frequency, mean and standard deviation. To obtain homogenous groups at baseline, differences between groups were analyzed by χ2 and *t*-test and GSES data were analyzed by Minitab software using repeated data analyzing model with the following formula:

$$\begin{array}{l} \mathrm{Scale}\;\mathrm{Variation}=\mathrm{Personal}\;\mathrm{difference}+\mathrm{Time}\\ \;+\mathrm{Group}\;\mathrm{in}\;\mathrm{time}+\mathrm{Residuals} \end{array}$$

## Results

All patients (n = 80) were randomized into two groups of control (n = 40) and case (n = 40).

The demographic and self-efficacy characteristics of both groups were similar at baseline. The mean age of the patients was 57.05 ± 1.51 and 57.78 ± 1.36 years in case and control groups respectively and *T*-test showed no statistically significant differences between groups (P = 0.624). Maximum and minimum age of the participants were 76 was 40 years respectively. There were 29 (72.5%) and 31 (77.5%) males in case and control groups respectively which was not statistically significant (P = 0.398).

There were no first time (before beginning of rehabilitation program) GESE differences between case (26.36 ± 0.84) and control (28.53 ± 0.54) groups (P = 0.44).

Analysis of repeated measurement data revealed that self-efficacy scale in repeated measurements during the time improved and there were statistically significant differences between two studied groups; i.e. our intervention affected this scale resulting in improved self-efficacy and better results in the patients of case group (P =0.003) (Table 1). However, there was no significant difference at different times (P =0.081); the scale did not improve in time.

**Table 1:** Self efficacy trend of patients continued program at home in compare with control

***Group***	***Time***	***Case***	***Control***
		***N = 40***	***N = 40***
***Time 1***		28.53 ± 0.94	26.36 ± 0.84
		(Mean ± SD)	(Mean ± SD)
***Time 2***		32.23 ± 0.90	24.19 ± 1.11
		(Mean ± SD)	(Mean ± SD)
***Time 3***		30.29 ± 1.11	27.16 ± 0.91
		(Mean ± SD)	(Mean ± SD)
***Time 4***		36.59 ± 5.65	26.5 ± 0.91
		(Mean ± SD)	(Mean ± SD)
***Result***		PV group = 0.003	
		PV Time =0.081	

*Time 1: beginning of program, Time2: finishing the program in rehabilitation center, Time 3: end of the first month, Time 4: end of the second month.

## Discussion

It has been found that a strong sense of personal efficacy is related to better health, higher achievement, and more social integration. A person who believes in being able to cause an event can conduct a more active and self-determined life course. Self-efficacy makes a difference in how people feel, think and act. In terms of feeling, a low sense of self-efficacy is associated with depression, anxiety, and helplessness. Self-efficacy levels can enhance or impede the motivation to act. Individuals with high self-efficacy choose to perform more challenging tasks. Perceived self-efficacy represents the belief that one can change risky health behaviors by personal action, e.g., by employing one’s skills to resist temptation. Behavioral changes are seen as dependent on one’s perceived capability to cope with stress and boredom and to mobilize one’s resources and courses of action required to meet the situational demands. Efficacy beliefs affect the intention to change risk behavior, the amount of effort expended to attain this goal, and the persistence to continue striving in spite of barriers and setbacks that may undermine motivation [18].

Previous studies have shown that women and elder patients in comparison with men and younger patients are less likely to participate in the program [19,20]. In the present study, a statistically significant difference was observed regarding self- efficacy scale between groups; in case group, nurse education, follow-up and home visits as well as continuing program at home affected self-efficacy resulting in improved scales. Dishman et al. determined that education of self-care strategies to patients with chronic conditions increases self-efficacy which in turn influences patients’ physical activity and disease complication; these findings are in line with our study (2008). In Wong study, the effect of nurse telephone follow-up on COPD patients was surveyed. It was determined that self-efficacy of patients who were followed-up significantly increased compared with the control group (P = 0.001) [21]; the findings of this study are in line with our findings as well.

### Limitations

This study only evaluated the effects which rehabilitation program has on patients’ self-efficacy. Therefore, furthers studies to reveal other effects of home-based cardiac rehabilitation such as well-being, return to job, further complications and other outcomes are recommended.

## Conclusion

Considering the findings of this study, home-based rehabilitation program has a positive effect on patients’ self-efficacy. Also these results can confirm that appropriate and effective training of patients, continuity of care and providing home follow-up can relieve the difficulties caused by patients not referring. Community health nurses play an effective role in the three levels of prevention. Performing home visits also is one of their most important and fundamental tasks. According to the available descriptions, community health nurses are the best options for continuing education, accompanying patients and performing follow-up at home.

## Competing interests

The authors have no competing interest.

## Authors’ contributions

SP, First Author, Conducting the proposal, Performing the program, Final preparation of the manuscript; AB, Helping to take samples, Scientific supervision of program; SG, Helping to take samples, Scientific supervision of program; AS, Helping in preparing proposal, Data analysis; SBA, Corresponding Author,Supervision the program, Helping to take samples and preparation of the manuscript. All authors read and approved the final manuscript.
